# The Certificate Nuisance

**Published:** 1895-06-22

**Authors:** 


### THE CERTIFICATE NUISANCE.

By the action of certain school boards and sanitary authorities a question is brought to a head which, from its importance both to the public and the profession, ought to be definitely settled on broad general principles. As it stands at present the position is this. Certain sanitary authorities refuse to recognise the certificates of the medical men who attend their employés when ill, and demand that when they stay off work more than two days they shall be visited by the medical officer of health, who shall investigate their cases and report whether or no they are *bonâ-fide* unfit for work. Various school boards also, being dissatisfied with the certificates received from medical men explaining the non-attendance of sick children, have appointed medical examiners, who, when any question arises in the mind of the school superintendent as to the sufficiency of a certificate, are to visit the case and report.

From the professional side the difficulty in both cases is the same, and arises from the fact that it is a universally recognised rule that no medical man shall interfere in any way with a patient under the care of another doctor. There can be no doubt that any medical man who visits a patient whom he knows to be under the care of another qualified practitioner, which he must know to be the case when a certificate has been received from him, commits a grave breach of a professional rule which is accepted all the world over; and it is equally certain that no resolution which any sanitary committee or other public body may choose to pass can have the slightest effect in lessening his iniquity in the eyes of his brethren. Under these circumstances it is not to be expected that practitioners of good standing can be obtained for the work, and it is an outrage that the certificates granted by the great mass of respectable general practitioners of London should be subjected to revision by men who, by accepting the task, show themselves to be outside the pale of professional honour.

If any medical officer of health has, in his arrangement with his sanitary authority, undertaken to attend their workpeople, by all means let him do so; and so long as he is attending he can give as many certificates as they like to require; but if he does not attend he cannot certify—that must be done by the man actually in attendance, who knows what is the matter, otherwise the whole affair becomes a farce.

As regards the school board certificates the matter is different, and in some respects much more difficult. On the one hand, the authorities wish to know whether the children who stay away from school are really ill, and want to appoint an examiner because they distrust the certificates received. On the other hand, practitioners not unnaturally consider that certificates which the Board, for their own purposes, compel the parents to ask for, should at least be accepted without cavil.

The fact is, that the doctors in giving the certificates are really doing the work of the school board visitors for nothing. We are quite prepared to admit that this is an unsatisfactory arrangement, and there are but few doctors who would not be thankful to see the whole system of certificate-giving put an end to, but so long as certificates are asked for they certainly should be received. If medical men give false certificates that is another matter; let them be prosecuted. If it is true, as was stated in the discussion at the last meeting of the School Board, that doctors have given certificates in regard to children whom they have not seen, or that there are places where "they could obtain as many certificates as they chose at a penny apiece," then it certainly is desirable that legal steps should be taken at once to purge the profession of such immorality, for the certificate system is too valuable to the poor to be lightly destroyed, as it will be if properly signed certificates are subjected to revision. No one who has not practised largely among the poor, and especially among the provident and careful members of the lower middle class, can have any idea of the extent to which the thrift agencies, the benefit clubs, and the various provident and sick societies make use of the doctor as a watch-dog over their funds. The constant giving of certificates for all sorts of purposes is a thankless task, undertaken by the profession wholly for the benefit of the poorer portion of the community, and it is difficult to see what agency could take its place. It is obvious, however, that the whole of this system will topple over like a house of cards if it is once officially maintained that a certificate duly signed by a properly qualified man is not to be accepted until it has been revised by an official. So called "practical people" are apt to take curiously small views, and, no doubt, while sanitary authorities look at the matter from one side, school boards look at it from the other, neither of them caring two pins for any aspect of the case except their own. These are, however, only fragments of the large question, and the broad principle on which this should be decided is that the sanctity of the medical certificate should be maintained, the full responsibility of those who sign it should be enforced, and all offenders in this respect should be unflinchingly prosecuted, but that then the medical certificate, properly signed by a qualified man, should be accepted without cavil.

